# Three new species of *Cataglyphis* Foerster, 1850 (Hymenoptera, Formicidae) from Iran

**DOI:** 10.3897/zookeys.1009.59205

**Published:** 2021-01-04

**Authors:** Arsalan Khalili-Moghadam, Sebastian Salata, Lech Borowiec

**Affiliations:** 1 Plant Protection Department, Agricultural College, Shahrekord University, Shahrekord, Iran Shahrekord University Shahrekord Iran; 2 Department of Biodiversity and Evolutionary Taxonomy, University of Wrocław, Przybyszewskiego 65, 51–148 Wrocław, Poland University of Wrocław Wrocław Poland; 3 California Academy of Sciences, 55 Music Concourse Dr, San Francisco, CA 94118, USA California Academy of Sciences San Francisco United States of America

**Keywords:** Central-western Iran, eastern Mediterranean, key to species, taxonomy

## Abstract

*Cataglyphis bazoftensis***sp. nov.**, *C. fritillariae***sp. nov.**, and *C. dejdaranensis***sp. nov.** are described from the Koohrang county of central-west Iran (Chaharmahal va Bakhtiari Province). All new species belong to the *C. altisquamis* complex and are characterized by presence of the thick, black, and decumbent setae on lateral and posterior surfaces of tibiae. Additionally, a key to Asian *Cataglyphis* was updated to accommodate the new species.

## Introduction

*Cataglyphis* Foerster, 1850 is a moderately large ant genus comprising 94 valid species and 18 valid subspecies divided into nine species groups (Agosti 1990; Bolton 2020). Its representatives are distributed mostly in the Palearctic Region’s semideserts and deserts (Agosti 1990; Brown 2000). However, *Cataglyphis* species were also recorded from other arid habitats, such as high altitude, mountain steppes, and forest steppes (Agosti 1990; Brown 2000). Members of the genus are among the commonest ants of the arid ecosystems of North Africa, Arabian Peninsula, and Central Asia, where they build crater nests directly in the ground (Agosti 1990; Collingwood and Agosti 1996; Brown 2000) and feed on dead insects (Lenoir et al. 2010). They have been reported as flower pollinators (Herrera et al. 1984) and as contributors in myrmecochorous seed dispersal (Hulme 1997; Boulay et al. 2007; Wehner 2020).

Many *Cataglyphis* species are polymorphic what causes challenges in their determination. Additionally, distinct polymorphism makes it necessary to prepare descriptions of new species based on nest samples. This approach should ensure comprehensive descriptions based on a detailed overview of infraspecific variability of studied samples. One should remember that genetic studies of some species did not confirm their homogeneity and suggested the presence of cryptic species and a high level of hybridization (Ionescu and Eyer 2016; Eyer et al. 2017).

The worldwide revision of the genus by Santschi (1929) is outdated, and the only relatively modern and global review of *Cataglyphis* was published three decades ago (Agosti 1990). On the regional level, the genus was studied for Arabian Peninsula (Collingwood and Agosti 1996; Sharaf et al. 2015), Armenia (Arakelian 1994), Bulgaria (Atanasov and Dlussky 1992), Northwest China (Chang and He 2002), Iraq (Pisarski 1965), Kingdom of Saudi Arabia (Collingwood 1985), Morocco (Cagniant 2009), Portugal (Collingwood and Prince 1998), Turkmenistan (Dlussky et al. 1992), former European U.S.S.R. (Arnol’di and Dlussky 1978), Asia (Radchenko 1997, 1998), Iberian Peninsula (Collingwood 1978), and Central and North Europe (Seifert 2018). Recent publications, which include changes in taxonomic status of some species and descriptions of new ones, show that the diversity of *Cataglyphis* is underestimated (Radchenko and Paknia 2010; Collingwood et al. 2011; Amor and Ortega 2014; Sharaf et al. 2015; Ionescu and Eyer 2016; Salata and Borowiec 2018).

Due to its location, geography, and predominance of open and arid habitats, Iran hosts the highest number of *Cataglyphis* worldwide (Paknia et al. 2008, Janicki et al. 2016). So far, there are 32 species of the genus known from the county, but some records need verification and confirmation (Paknia et al. 2008, 2009; Moradloo et al. 2015; Rad et al. 2018). The present work is a contribution to the understanding of the Iranian *Cataglyphis*. We describe three new species of the *C. altisquamis* species group (sensu Agosti 1990): *C. bazoftensis* sp. nov., *C. fritillariae* sp. nov., and *C. dejdaranensis* sp. nov. based on the worker caste. The *C. altisquamis* species group is characterized by the following combination of characters in the worker caste: relatively large (WL up to 5 mm); body dull, uniformly yellow-black to black or bicolored with black gaster, petiole cuneiform or pseudo-nodiform, head finely reticulate with punctulate frons. The geographic range of this group extends from Portugal and Morocco to Central Asia. Members of this group were included in Radchenko’s key (1998) to the Asian *Cataglyphis*. Herein, we modify the key to accommodate the new species. Additionally, we also included in the key *C. asiriensis* Collingwood, 1985, a member of the *altisquamis* species group known from Saudi Arabia, which was omitted by Radchenko (1998).

## Materials and methods

Investigated specimens were collected from five sites in the Koohrang County, located in the northern part of the Chaharmahal va Bakhtiari Province of Iran. All sites were placed at altitude from 1738 to 2778 m. a.s.l. The county is surrounded by the Zagros Mountains, one of the two largest mountain ranges of Iran, and is characterized by an alpine climate. The only exception is the Bazoft region, which is warmer than other parts of the Koohrang County and covered by deciduous oak forests.

The dominant sampling method was a direct sampling (hand collecting). Individual specimens were collected on the ground or from nests under stones. All specimens were preserved in 75% EtOH. Photos were taken using a Nikon SMZ 1500 stereomicroscope, Nikon D5200 photo camera, and Helicon Focus software. All given label data are in the original spelling, only the geographic coordinates are given in decimal notation, instead of the degrees, minutes, seconds on the labels; a vertical bar (|) separates data on different rows and double vertical bars (||) separate labels. Type specimens’ photographs are available online on AntWeb (www.AntWeb.org) and are accessible using the unique CASENT identifying specimen code.

Museum abbreviations (Evenhuis 2020):

**MNHW** Museum of Natural History, University of Wrocław, Poland, in temporary deposit in Department of Biodiversity and Evolutionary Taxonomy, University of Wrocław, Poland;

**MHNG**Muséum d’Historie Naturelle, Genève, Switzerland;

**USMB**Upper Silesian Museum, Bytom, Poland.

Pilosity inclination degree follows that used in Wilson (1955). Adpressed (0–5°) hairs run parallel or nearly parallel to the body surface. Decumbent hairs stand 10–40°, subdecumbent hair stands ~45° from the surface, suberect hairs bend about 10–20° from vertical, and erect hairs stand vertical or nearly vertical.

Measurements: all measurements are given in mm.

**HFL** hind femur length; measured on dorsal side from trochanter to apex of femur;

**HL** head length; measured in a straight line from mid-point of anterior clypeal margin to mid-point of posterior margin in full-face view;

**HW** head width; measured in full-face view directly behind the eyes;

**PRL** propodeum length; measured in lateral view, from metanotal groove to posterior-most point of propodeum;

**PRW** propodeal width; maximum width of propodeum in dorsal view;

**PTH** petiole height; the chord of ventral petiolar profile at node level is the reference line perpendicular to which the maximum height of petiole is measured, measured in lateral view;

**PTW** petiole width; maximum width of the petiolar node in lateral view;

**PW** pronotum width; maximum width of pronotum in dorsal view;

**SL** scape length; maximum straight-line length of scape excluding the basal condylar bulb;

**WL** Weber’s length; measured as diagonal length from the anterior end of the neck shield to the posterior margin of the propodeal lobe.

### Ratios

**CI** cephalic index, HL/HW;

**FI** femur index, HFL/WL;

**PI** petiole index, PTH/PTW;

**SI** scape index, SL/HL.

## Results

### Synoptic list of *Cataglyphis* of Iran

The list is created based on data from Paknia et al. (2008) and Janicki et al. (2016), while the species-group divisions follows Agosti (1990) and Radchenko and Paknia (2010).

***Cataglyphis albicans* species group**

*Cataglyphis albicans* (Roger, 1859)

First record from Iran: Ghahari et al. (2011).

*Cataglyphis alibabae* Pisarski, 1965

First record from Iran: Rad et al. (2018).

*Cataglyphis aurata* Menozzi, 1932

First record from Iran: Ghahari et al. (2009).

*Cataglyphis cinnamomea* (Karavaiev, 1910)

First record from Iran: Paknia et al. (2010).

*Cataglyphis cuneinodis* Arnol’di, 1964

First record from Iran: Radchenko (1998).

*Cataglyphis elegantissima* Arnol’di, 1968

First record from Iran: Radchenko (1998).

*Cataglyphis livida* (André, 1881)

First record from Iran: Forel (1904).

*Cataglyphis rubra* (Forel, 1903)

First record from Iran: Radchenko (1998).

*Cataglyphis semitonsa* Santschi, 1929

First record from Iran: Ghahari et al. (2009).

*Cataglyphis viaticoides* (André, 1881)

First record from Iran: Rad et al. (2018).

***Cataglyphis alitisquamis* species group**

*Cataglyphis altisquamis* (André, 1881)

First record from Iran: Paknia et al. (2008).

*Cataglyphis bazoftensis* sp. nov.

*Cataglyphis bucharica* Emery, 1925

First record from Iran: Radchenko (1998).

*Cataglyphis dejdaranensis* sp. nov.

*Cataglyphis foreli* (Ruzsky, 1903)

First record from Iran: Forel (1904).

*Cataglyphis fritillariae* sp. nov.

*Cataglyphis kurdistanica* Pisarski, 1965

First record from Iran: Paknia et al. (2010).

***Cataglyphis bicolor* species group**

*Cataglyphis abyssinica* (Forel, 1904)

First record from Iran: Ghahari and Collingwood (2011).

*Cataglyphis bellicosa* (Karavaiev, 1924)

First record from Iran: Karavaiev (1924).

*Cataglyphis bergiana* Arnol’di, 1964

First record from Iran: Paknia et al. (2010).

*Cataglyphis diehlii* (Forel, 1902)

First record from Iran: Ghahari and Collingwood (2011).

*Cataglyphis isis* (Forel, 1913)

First record from Iran: Crawley (1920).

*Cataglyphis longipedem* (Eichwald, 1841)

First record from Iran: Forel (1904).

*Cataglyphis nigra* (André, 1881)

First record from Iran: Forel (1904).

*Cataglyphis nodus* (Brullé, 1833)

First record from Iran: Radchenko (1998).

*Cataglyphis oasium* Menozzi, 1932

First record from Iran: Ionescu and Eyer (2016).

*Cataglyphis setipes* (Forel, 1894)

First record from Iran: Radchenko (1998).

*Cataglyphis stigmata* Radchenko & Paknia, 2010

First record from Iran: Radchenko and Paknia (2010).

***Cataglyphis cursor* species group**

*Cataglyphis aenescens* (Nylander, 1849)

First record from Iran: Forel (1904).

*Cataglyphis cugiai* Menozzi, 1939

First record from Iran: Paknia et al. (2010).

*Cataglyphis frigida* (André, 1881)

First record from Iran: Emery (1906).

*Cataglyphis frigida persica* (Emery, 1906)

First record from Iran: Emery (1906).

*Cataglyphis pubescens* Radchenko & Paknia, 2010

First record from Iran: Radchenko and Paknia (2010).

***Cataglyphis nigripes* species group**

*Cataglyphis nigripes* Arnol’di, 1964

First record from Iran: Radchenko (1998).

***Cataglyphis pallida* species group**

*Cataglyphis emeryi* (Karavaiev, 1911)

First record from Iran: Radchenko (1998).

## Taxonomy

### Cataglyphis bazoftensis sp. nov.

Taxon classificationAnimaliaHymenopteraFormicidae

47DFCAB7-32B4-5954-A1B4-B661D07C8686

http://zoobank.org/A586710F-F2AE-496E-91F3-93A454CF641E

Figs 1–2 , 3 , 4–6 , 7–8 , 27

#### Type material.

***Holotype***: major worker (CASENT0872262): IRAN, Chaharmahal Va | Bakhtiari, Koohrang (Bazoft) | 1 VII 2017, 1754 m | leg. Khalili-Moghadam | 32.2969 / 49.9358 || LBC | | LBC-IR00083 (MNHW); ***paratypes***: 12 major, 2 medium and 9 minor workers (CASENT0872263–CASENT0872285): the same data as holotype (MNHW, MHNG, USMB); 3 major, 2 medium and 4 minor workers (CASENT0872286–CASENT0872294): IRAN, Chaharmahal Va | Bakhtiari, Koohrang (Bazoft) | 1 VII 2017, 1798 m | leg. Khalili-Moghadam | 32.2927 / 49.9391 || Collection L. Borowiec || LBC-IR00082 (MNHW, MHNG); three major workers (CASENT0872295–CASENT0872297): IRAN, Chaharmahal Va | Bakhtiari, Koohrang (Bazoft) | 1 VII 2017, 1886 m | leg. Khalili-Moghadam | 32.4855 / 49.7472 || LBC | Formicidae | LBC-IR00079 (MNHW, MHNG); major worker (CASENT0872298): IRAN, Chaharmahal Va | Bakhtiari, Koohrang (Bazoft) | 1 VII 2017, 1738 m | leg. Khalili-Moghadam | 32.3766 / 49.8594 || LBC | Formicidae | LBC-IR00079 (MNHW).

**Figures 1, 2.**
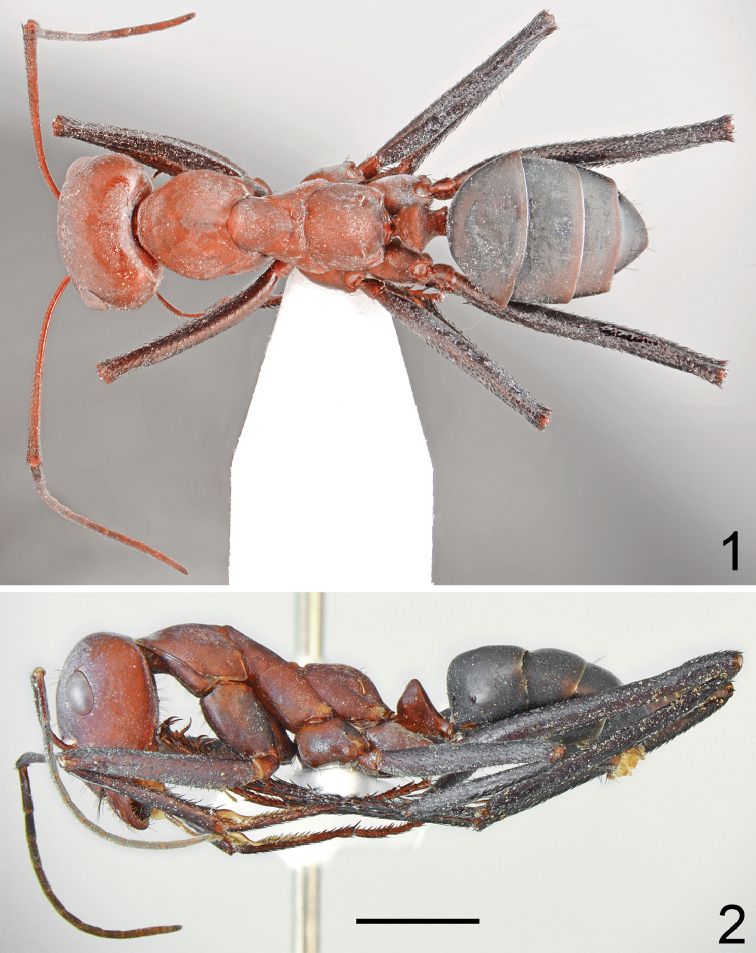
Major worker of *Cataglyphis bazoftensis* sp. nov. **1** dorsal **2** lateral. Scale bar 2 mm.

#### Diagnosis.

Bicolored body combined with thick, dense, black, decumbent setae on femora and tibiae resemble characters typical for species of the *Cataglyphis setipes* complex. *Cataglyphis bazoftensis* distinctly differs from them in the cuneiform petiolar node, which is distinctly nodiform among members of the *setipes* complex. At first glance, large species of the *Cataglyphis bucharica* complex, with cuneiform petiole, resemble *C. bazoftensis*, but most of these species differ in femora and tibiae lacking thick, black, decumbent setae. Members of the *bucharica* complex with setose femora and tibiae differ in the presence of black, erect setae on the posterior part of the head. *Cataglyphis kurdistanica* Pisarski is the most similar to *C. bazoftensis*, but differs in the presence of the major soldier caste, and posterior part of head and propodeum covered with long, black and erect setae. *Cataglyphis altisquamis* (André) and *C. foreli* (Ruzsky) differ from *C. bazoftensis* in major workers with reddish brown to brownish black head and mesosoma; while *C. bazoftensis* has major workers with uniformly red head and mesosoma (only the smallest majors of *C. bazoftensis* can be sometimes reddish brown).

#### Description.

**Figure 3.**
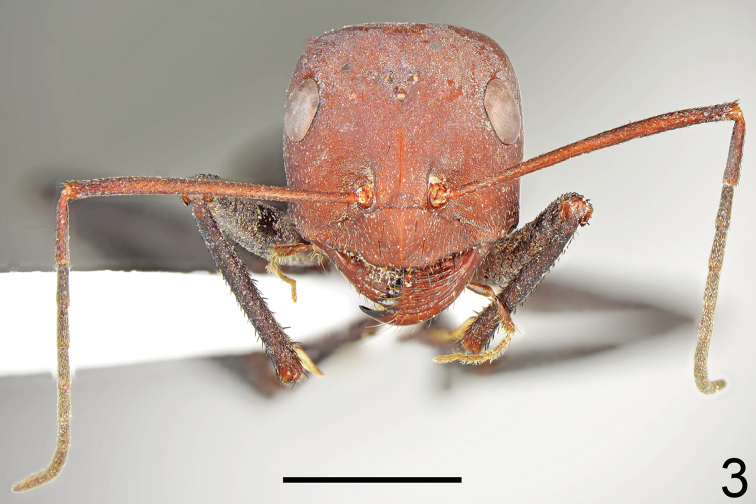
Major worker of *Cataglyphis bazoftensis* sp. nov., head and antennae. Scale bar 2 mm.

Major worker (n = 10): ***Measurements.***HL: 2.945 (2.72–3.04); HW: 2.738 (2.42–2.88); SL: 3.600 (3.39–3.80); PW: 1.923 (1.77–2.05); PRL: 1.732 (1.59–1.78); PRW: 1.457 (1.27–1.58); PTH: 0.955 (0.81–1.05); PTW: 0.945 (0.87–1.00); WL: 4.580 (4.25–4.78); HFL: 5.308 (5.12–5.58); CI: 1.077 (1.056–1.124); SI: 1.223 (1.184–1.246); PI: 1.011 (0.931–1.114); FI: 1.160 (1.105–1.205). ***Color.*** Head, mesosoma, and petiolar node red in the largest major workers; the percentage of brown in body coloration increases in smaller major workers with the smallest major workers reddish brown. Legs most often black or black with brownish black coxa; in the largest major workers coxa and trochanters mostly reddish brown to brown with darker brown spots of diffused borders; in the palest specimens femora brownish black with reddish iridescence, tibiae sometimes reddish brown apically, and tarsi reddish brown to brown. Antennal scapus red to red-brown; only in the darkest major workers brown; funicles darker than scapus, from brown to almost black, only in the palest specimens red-brown (Fig. 3). ***Head.*** Square; approximately 1.05× as long as wide; sides below eyes almost parallel, above eyes gently convex; posterior margin of head almost straight (Fig. 3). Anterior clypeal margin convex; without central impression; clypeal plate with three or four long black central setae and a pair of moderately long basal setae; anterior clypeal margin with a row of short black setae, and 8–10 long black setae, the longest as long as 0.75 length of clypeus; some workers have long setae often worn off or broken. Clypeus shiny and densely microreticulate; covered with very sparse and short, appressed pubescence. Eyes large and oval; approximately 1.3–1.4× as long as wide. Frontal carinae short; not extending beyond frontal lobes; interocular area with thin and shiny line and a pair of long black setae. Antennal fossa shallow; opalescent and densely microreticulated. Head shiny and densely microreticulate; mostly without appressed pubescence; only antennal fossa, posterior margin of head, and gular parts covered with sparse, short, adpressed hairs. Ocellar region with 2–4 moderately long and black setae; posterior angles without black setae; rest of frontal and lateral faces of head without erect setae; ventral side with a dozen yellowish to brown setae; interocular and ocular setae often broken. Antennal scape long; in frontal view almost straight, 1.3× as long as width of the head; base without tooth; apex slightly and gradually widening; funiculus long; pedicel elongated, approximately 0.82× as long as segments II and III combined, 1.6× as long as segment II (Fig. 3). Surface of scape densely microsculptured; distinctly to moderately shiny; covered with thick, moderately dense and decumbent setae. Mandibles rounded; basally smooth and shiny; apical ¾ length with deep grooves; surface shiny with several long yellow setae; masticatory margin with four large teeth. ***Mesosoma.*** Long; 2.4× as long as wide; metanotal groove shallow (Fig. 2). Pronotum convex on sides (Fig. 1). In lateral view promesonotum slightly arched in profile; propodeum positioned lower than promesonotum, moderately convex in lateral view (Fig. 2). Mesosoma shiny and densely microreticulated; covered with sparse, short, appressed pubescence; lateral sides of pronotum and mesonotum almost hairless; anterior part of pronotum, posterior angles of both mesonotum and propodeum with indistinct vestiture. Pronotum and mesonotum without erect setae; propodeum without or with one or two short, black, erect setae. ***Petiole.*** Cuneiform; in lateral view almost triangular with very short peduncle. Anterior face in front of spiracle distinctly convex; posterior face almost flat; top of petiole in lateral view obtusely angulate, lacking erect setae; sometimes frontal face apically with a single short seta. In anterior or posterior view petiolar dorsum emarginated. Surface of petiole distinctly microreticulated; shiny to slightly opalescent. ***Gaster.*** Dull and distinctly microreticulate. Whole surface of gaster with indistinct, sparse, appressed pubescence; tergites I and II without erect setae; tergite III with 2–4 long, black, central setae placed close to anterior margin, and none or one seta at lateral margin; tergite IV in with 6–8 long black setae; setosity in older specimens usually broken (Fig. 2). Each of gastral sternites with three or four long, black, erect setae. ***Legs.*** Dorsal and lateral surfaces of femora and tibiae covered with thick, dense, black, decumbent setae; interspaces between setae hairless. Ventral surfaces of femora and tibiae with numerous long, black, suberect to erect, spiniform setae.

Minor worker (n = 10): ***Measurements.***HL: 1.308 (1.27–1.41); HW: 1.138 (1.09–1.22); SL: 1.270 (1.20–1.43); PW: 0.815 (0.76–0.88); PRL: 0.738 (0.68–0.82); PRW: 0.593 (0.55–0.65); PTH: 0.507 (0.47–0.56); PTW: 0.363 (0.34–0.40); WL: 1.957 (2.02–2.48); HFL: 1.697 (1.59–1.86); CI: 1.151 (1.142–1.174); SI: 0.970 (0.930–1.014); PI: 1.396 (1.342–1.559); FI: 0.867 (0.834–0.883).

***Color*.** Head and mesosoma uniformly brown to black or brown with diffused red-brown parts; gaster, petiole, femora, and tibiae brown; trochanters and tarsi yellow-brown. Younger specimens often paler than older specimens, with large areas of body yellowish brown. Antennae bright brown, only in younger specimens mostly yellowish to yellowish brown (Figs 7, 8). ***Head*.** More elongated than in major workers; 1.2× as long as wide; below eyes parallel sided, behind eyes regularly rounded; posterior margin of head convex. Anterior clypeal margin convex with shallow impression in central part. Eyes large and oval; 1.3–1.4× as long as wide. Sculpture and setation of head and legs same as in major worker. ***Mesosoma*.** Same as in major worker. ***Petiole*.** More conical than cuneiform; dorsum more rounded, anterior face slightly convex (Fig. 5). ***Gaster*.** Strongly microreticulated and dull. Tergites I and II without erect setae; tergite III with only a pair of black setae centrally; tergite IV with two long, and two short black setae; in older specimens, setae usually partially broken (Fig. 8). Each of gastral sternites with 2–4 long, black, erect setae.

**Figures 4–6.**
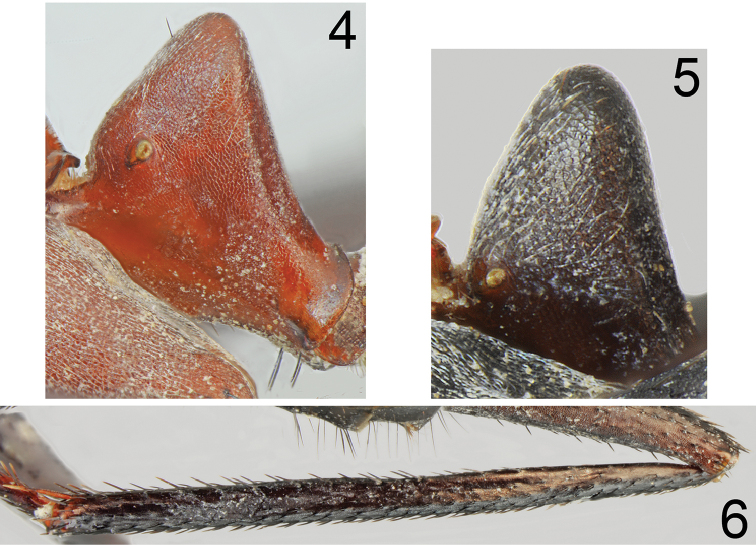
*Cataglyphis bazoftensis* sp. nov. **4** petiole of major worker **5** petiole of minor worker **6** hind tibia (not to scale).

#### Biology.

Little known, nests were found under stones inside a deciduous, oak forest surrounded by a grazing area. All collecting sites were in alpine zone, from 1738 to 1886 m a.s.l.

#### Etymology.

The species name *bazoftensis* is a feminine Latin adjective in the nominative case and refers to the Bazoft region of the Koohrang County, the type locality for this species.

**Figures 7, 8.**
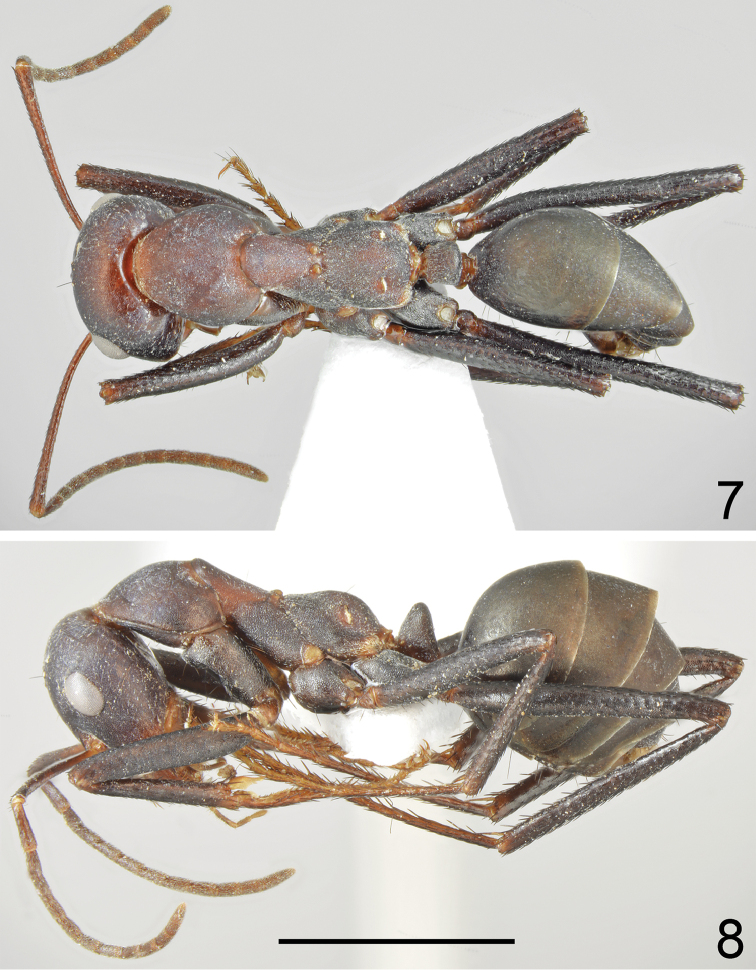
Minor worker of *Cataglyphis bazoftensis* sp. nov. **7** dorsal **8** lateral. Scale bar 2 mm.

### Cataglyphis fritillariae sp. nov.

Taxon classificationAnimaliaHymenopteraFormicidae

527C9FD8-8C85-5B6C-885D-6FD0EC2DC73A

http://zoobank.org/25117B97-D60C-4746-98AA-816D3C1259C3

Figs 9–10 , 11 , 12–14 , 15–16 , 28

#### Type material.

***Holotype***: major worker (CASENT0872299): IRAN, Chaharmahal Va | Bakhtiari, Koohrang | Dashte laleh, 2400 m || 32.5884 / 50.2002 | 25 V 2017 | A. Khalili-Moghadam || Collection L. Borowiec | Formicidae | LBC-IR00069 (MNHW); ***paratypes***: 16 major, 11 medium and 43 minor workers (CASENT0872300-CASENT0872369): the same data as holotype (MNHW, MHNG, USMB).

#### Other material.

Two major and two minor workers: IRAN, Chaharmahal Va | Bakhtiari, Koohrang (Dashte | laleh), 25 IV 2017, 2400 m | leg. Khalili-Moghadam | 32.5886 / 50.2002 || Collection L. Borowiec | Formicidae | LBC-IR00084 (MNHW); 7 major workers: IRAN, Chaharmahal Va | Bakhtiari, Koohrang (Dashte | laleh), 25 IV 2017, 2391 m | leg. Khalili-Moghadam | 32.5875 / 50.2002 || Collection L. Borowiec | Formicidae | LBC-IR00086 (MNHW); two major workers: IRAN, Chaharmahal Va | Bakhtiari, Koohrang (Soodejan) | 25 IV 2017, 2143 m | leg. Khalili-Moghadam | 32.5425 / 50.3505 || Collection L. Borowiec | Formicidae | LBC-IR00087 (MNHW); major worker: IRAN, Chaharmahal Va | Bakhtiari, Koohrang (Dashte | laleh), 25 IV 2017, 2400 m | leg. Khalili-Moghadam | 32.5886 / 50.2002 || Collection L. Borowiec | Formicidae | LBC-IR00078 (MNHW).

**Figures 9, 10.**
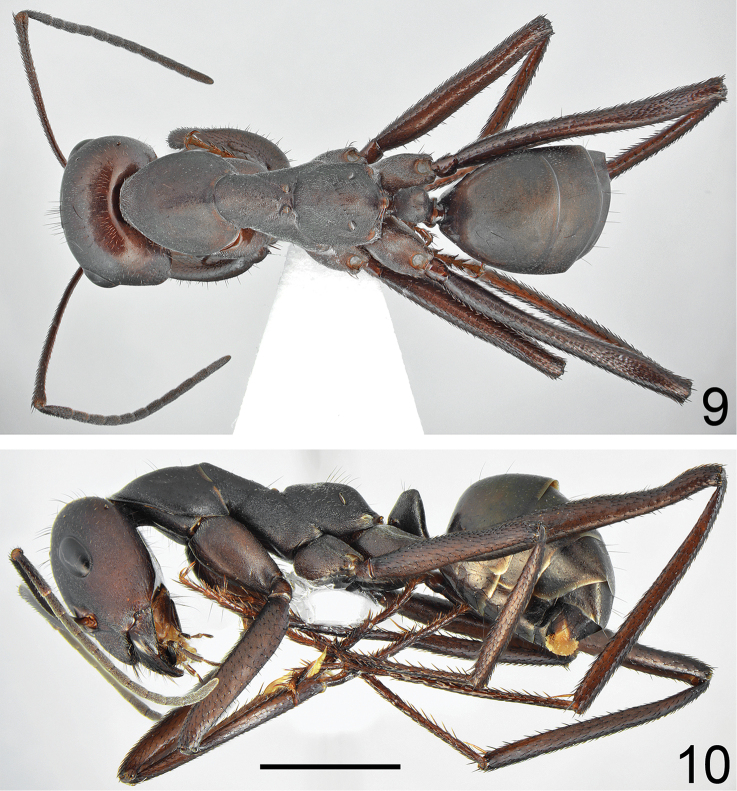
Major worker of *Cataglyphis fritillariae* sp. nov. **9** dorsal **10** lateral. Scale bar 2 mm.

#### Diagnosis.

*Cataglyphis fritillariae* belongs to a group of large species with well-developed and dull body sculpture. Femora and tibiae covered with thick, dense, black, decumbent setae cluster this species with the *Cataglyphis setipes* complex, while the shape of the petiolar node groups it with the *C. altisquamis* complex. At first glance, *C. foreli* appears similar to *C. fritillariae*, but it differs by the trapezoidal profile of petiole, larger eyes, and lack of thick, black, decumbent setae on femora and tibia. *Cataglyphis kurdistanica* Pisarski has similar petiole and black decumbent setae on legs, but it can be separated by the bicolored body and the presence of a soldier caste. *Cataglyphis bucharica* also appears similar to *C. fritillariae*, but it is readily recognized by reddish head and mesosoma, presence of numerous erect setae on propodeum, longer propodeal spiracle, and absence of thick, black, decumbent setae on femora. *Cataglyphis asiriensis* Collingwood, known from the Asir Mountains (Kingdom of Saudi Arabia), has a similar petiole shape and the legs covered with black decumbent setae but it differs from *C. fritillariae* by the presence of long, black, erect setae also present on the dorsal side of femora and tibiae, and more numerous black erect setae on the mesosoma. *Cataglyphis dejdaranensis* sp. nov. is the most similar to *C. fritillariae*, but it differs by the weakly sculptured gaster that has moderately shiny sides of gastral tergite I, petiolar node of major workers knob-shaped in profile, and more convex propodeum.

#### Description.

**Figure 11.**
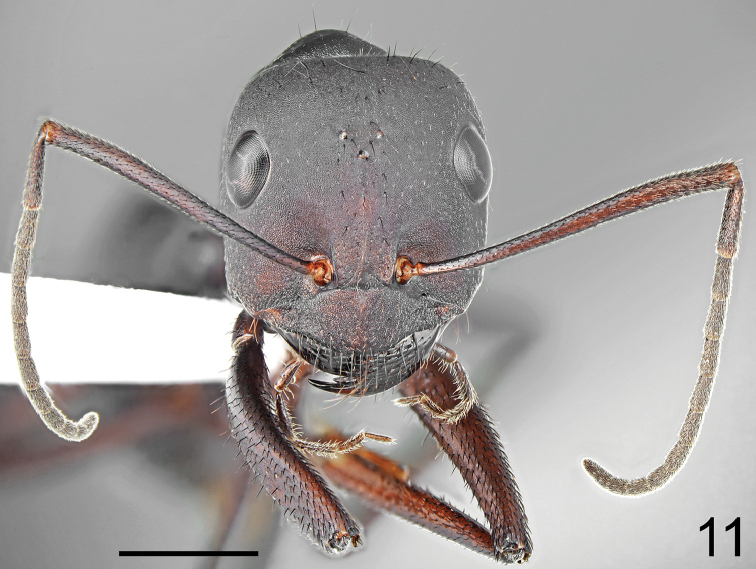
Major worker of *Cataglyphis fritillariae* sp. nov., head and antennae. Scale bar 1 mm.

**Figures 12–14.**
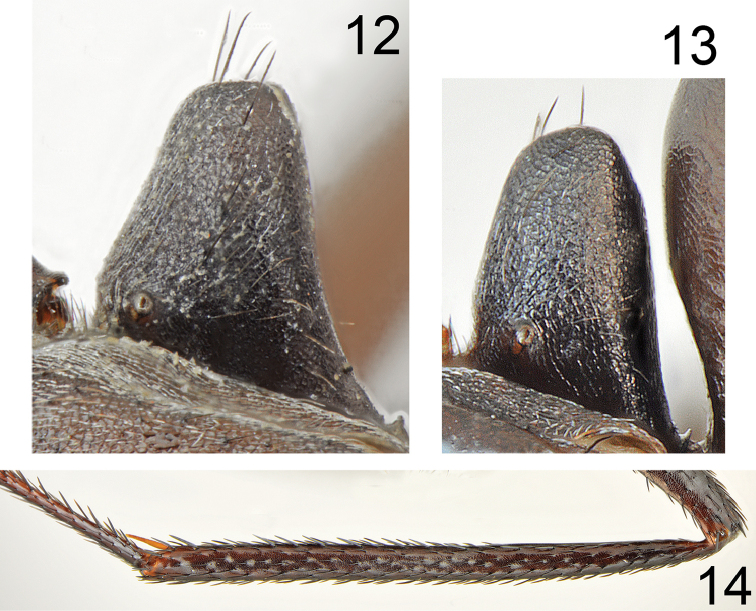
*Cataglyphis fritillariae* sp. nov. **12** petiole of major worker **13** petiole of minor worker **14** hind tibia (not in scale).

Major worker (n = 15): ***Measurements.***HL: 2.435 (2.31–2.55); HW: 2.203 (2.10–2.33); SL: 2.732 (2.63–2.90); PW: 1.582 (1.50–1.69); PRL: 1.430 (1.35–1.52); PRW: 1.155 (1.07–1.260); PTH: 0.878 (0.78–1.11); PTW: 0.715 (0.66–0.78); WL: 3.777 (3.63–3.92); HFL: 4.050 (3.76–4.27); CI: 1.105 (1.064–1.123); SI: 1.122 (1.113–1.137); PI: 1.292 (1.164–1.423); FI: 1.072 (1.019–1.109). ***Color.*** Head, mesosoma and gaster uniformly black or black with indistinct brownish black spots with diffused borders. Legs uniformly black to brownish black. Antennae completely black or black with brownish black scape (Figs 9–11). ***Head.*** Square; approximately 1.13× as long as wide; sides below eyes almost parallel, above eyes gently convex, posterior margin almost straight (Fig. 11). Anterior clypeal margin convex; without central impression; with a row of short black setae, and eight additional long black setae, the longest approximately as long as 0.6 length of clypeus; clypeal plate with a pair of long and black setae close to basal margin, and two pairs of similar setae close to anterior margin; sometimes clypeal plate with 1–3 additional short setae. Clypeus opalescent and densely microreticulated; covered with very sparse, short and adpressed hairs. Eyes large and oval, 1.3–1.4× as long as wide. Frontal carinae short; not extending beyond frontal lobes; interocular area with thin shiny line and 1–3 long black setae placed along each side of the line. Antennal fossa shallow, opalescent, densely microreticulated. Head opalescent and densely microreticulated; covered with sparse, short, adpressed hairs (Fig. 11). Ocellar region with three or four moderately long and black setae and often additional two or three shorter black setae; a transverse row of 4–8 black setae present above ocelli; area behind eyes with three or four yellowish to brown short setae; rest of frontal and lateral faces of head without erect setae; ventral side of the head with a dozen white to brown setae. Antennal scape long; in frontal view straight; 1.3× as long as width of the head; from base to apex slightly and gradually widened; its base without tooth; funiculus long; pedicel elongated, approximately 0.96× as long as segments II+III combined and 1.9× as long as segment II (Fig. 11). Surface of scape densely microsculptured and opalescent; covered with thick, dense, decumbent setae. Mandibles rounded; basally smooth and shiny; apical ¾ length with deep grooves; surface shiny with several long, white to brown setae; masticatory margin with four large teeth. ***Mesosoma.*** Long; 2.4× as long as wide; metanotal groove shallow (Fig. 10). Pronotum convex on sides. In lateral view promesonotum slightly arched in profile; propodeum positioned lower than promesonotum, moderately convex in lateral view (Fig. 10). Mesosoma opalescent and densely microreticulated; covered with sparse, short and adpressed hairs. Pronotum with 2–5 moderately long and black setae medially, sometimes with one or two short black setae close to its anterior and posterior margins; mesonotum with one or two moderately long and black setae in front of spiracles, sometimes with one or two short, black setae medially and anteriorly; propodeum dorsally with 4–7 long and black setae, and often two or three additional short setae; older specimens sometimes with setae completely to partly broken (Fig. 10). ***Petiole.*** Trapezoidal in profile; its anterior face mostly flat with only basal part convex; posterior face straight to slightly concave; dorsum flat; peduncle very short. Surface opalescent and microreticulated; covered with sparse, short and adpressed hairs; top of knob with 3–6 moderately long, black, erect setae (Fig. 12). In anterior view petiolar dorsum with distinct emargination medially. ***Gaster.*** Dull and distinctly microreticulated. Whole surface of gaster with short, sparse, adpressed hairs; tergite I in anterior part with 2–4 long, black setae; tergite II without a pair of black setae anteriorly; tergite III with 2–4 long and black setae centrally; in older specimens, setae usually broken (Fig. 16). Each of gastral sternites with three or four long, black, erect setae. ***Legs.*** Dorsal and lateral surfaces of femora and tibiae covered with thick, dense, black, decumbent setae; no white, adpressed setae on surface between black setae. Ventral surfaces of femora and tibiae with numerous long, black, suberect to erect, spiniform setae (Fig. 14).

Minor worker (n=15): ***Measurements.***HL: 1.240 (0.98–1.52); HW: 1.083 (0.87–1.32); SL: 1.168 (0.84–1.57); PW: 0.753 (0.59–0.92); PRL: 0.692 (0.51–0.88); PRW: 0.552 (0.44–0.69); PTH: 0.518 (0.40–0.61); PTW: 0.330 (0.27–0.45); WL: 1.818 (1.39–2.34); HFL: 1.573 (1.10–2.20); CI: 1.145 (1.126–1.173); SI: 0.935 (0.857–1.033); PI: 1.606 (1.356–2.000); FI: 0.858 (0.791–0.940).

**Figures 15, 16.**
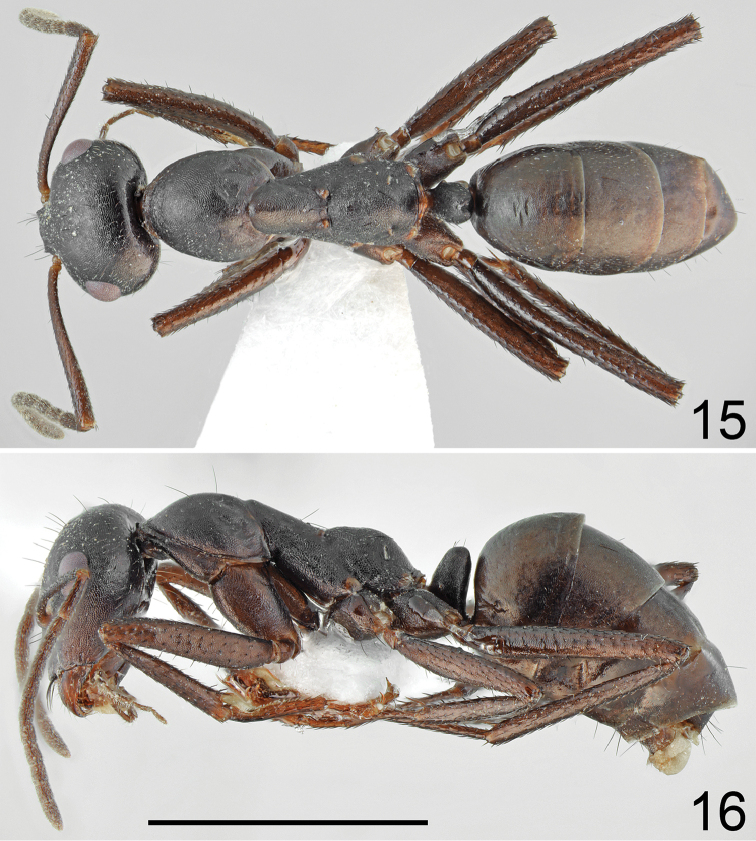
Minor worker of *Cataglyphis fritillariae* sp. nov. **15** dorsal **16** lateral. Scale bar 2 mm.

***Color.*** Uniformly yellowish brown to brownish black. Antennae and legs yellowish brown to bright brown (Figs 15, 16). ***Head.*** Almost square; 1.15–1.17× as long as wide; sides below eyes almost parallel, behind eyes regularly convex, posterior margin of head convex. Sculpture and setation of the head similar as in major worker but with lower number of long setae. ***Mesosoma.*** Long; 2.4–2.5× as long as wide; metanotal groove shallow. Pronotum convex on sides. In lateral view promesonotum slightly arched in profile; propodeum positioned lower than promesonotum, its dorsum and posterior side slightly convex (Fig. 16). Whole mesosoma opalescent and densely microreticulated (Figs 15, 16). Whole mesosoma covered with dense, short, adpressed hair; pronotum with additional two or three black and erect setae; mesonotum and propodeum with additional one or two black and erect setae. ***Petiole.*** In form of thick scale; its anterior surface slightly convex; apex rounded and posterior surface almost flat; surface microreticulated and covered by sparse, short, adpressed hairs; dorsum with 1–3 black, moderately elongated and erect setae (Fig. 13). ***Gaster.*** Dull and distinctly microreticulated; tergites I and II with up to two black and erect setae; tergite III without or with a pair of black and erect setae close to its anterior margin; tergite IV with 2–4 erect setae; sternites with two or three black and erect setae; whole surface of gaster with short, adpressed hairs (Figs 15, 16). ***Legs.*** Dorsal and lateral surfaces of femora and tibiae covered with thick, dense, black, decumbent setae; no white, adpressed setae on surface between black setae. Ventral surfaces of femora and tibiae with numerous long, black, suberect to erect, spiniform setae.

#### Biology.

Little known, in Dashte laleh a nest was found under rocks in a grazing area (Fig. 25). The site was located on a small plateau (3600 hectares), between 2100–2600 m above sea level, and in May was predominantly overgrown by Snake’s head (*Fritillaria imperialis* L.). Other common plants recorded from this locality were Milkvetch (*Astragalus* spp.), Persian shallot (*Allium stipitatum* Regel), and kheshk (*Daphne mucronata* Royle). The species appears to be alpine, as all its collecting sites were placed on high altitude from 2143 to 2400 m.

#### Etymology.

The species name *fritillariae* is named after the genitive singular case of the generic name of the Snake’s head *Fritillaria imperialis* L., the dominant flower in Dashte laleh, the type locality of this ant species.

### Cataglyphis dejdaranensis sp. nov.

Taxon classificationAnimaliaHymenopteraFormicidae

2B38ACA9-B8E9-5EDC-BFE7-E19EF3CFB2CD

http://zoobank.org/31D1EF27-49CB-4218-8A8A-AD5706CF4421

Figs 17–18 , 19 , 20–22 , 23, 24 , 25–26

#### Type material.

***Holotype***: major worker (CASENT0872370): IRAN, Chaharmahal Va | Bakhtiari, Koohrang (Cheri) | 2 VI 2017, 2778 m | leg. Khalili-Moghadam | 32.1686 / 50.1752 || Collection L. Borowiec | Formicidae | LBC-IR00088 (MNHW); ***paratypes***: one major and two minor workers (CASENT0872371–CASENT0872373): the same data as holotype (MHNG, MNHW); ***paratypes***: four major and two minor workers (CASENT0872374–CASENT0872379): IRAN, Chaharmahal Va | Bakhtiari, Koohrang (Dejdaran | valley) 2 VI 2017, 2319 m | leg. Khalili-Moghadam | 32.1955 / 50.2075 || Collection L. Borowiec | Formicidae | LBC-IR00076 (MNHW).

**Figures 17, 18.**
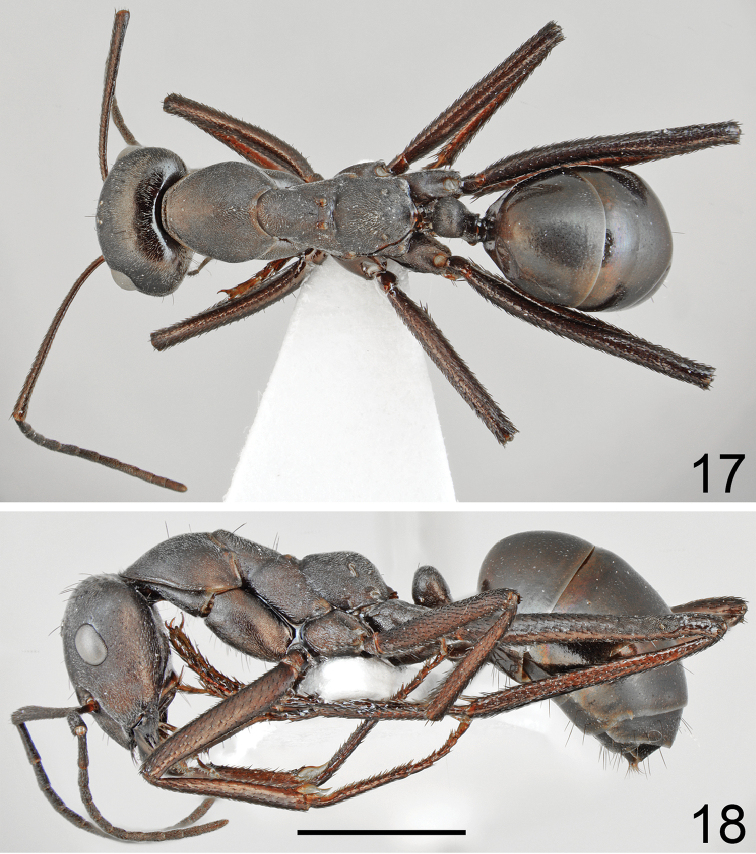
Major worker of *Cataglyphis dejdaranensis* sp. nov. **17** dorsal **18** lateral. Scale bar 2 mm.

#### Diagnosis.

*Cataglyphis dejdaranensis* belongs to the group of large species with well-developed and dull body sculpture. Femora and tibiae covered with thick, dense, black, decumbent setae cluster this species with the *Cataglyphis setipes* complex, and the shape of the petiolar node groups it with the *C. altisquamis* complex. From all species of the *C. setipes* complex, *C. dejdaranensis* differs in a knob-shaped petiole, which is not forming a spherical node; from all species of the *C. altisquamis* complex, *C. dejdaranensis* differs in a weak microsculpture of gaster of which at least sides are visibly shiny. *Cataglyphis dejdaranensis* has the least sculpted gaster within all large species with well-developed body microsculpture. At first glance, *C. foreli* appears similar to *C. dejdaranensis*, but it differs in larger eyes, and femora and tibia lacking thick, black, decumbent setae. *Cataglyphis kurdistanica* Pisarski has similarly shaped petiole and legs with black decumbent setae, but it differs in bicolored body and presence of the soldier caste. *Cataglyphis bucharica* also appears similar to *C. dejdaranensis* but it differs in reddish head and mesosoma, numerous erect setae on propodeum, longer propodeal spiracle, and femora and tibia lacking thick, black, decumbent setae. *Cataglyphis fritillariae* sp. nov. is the most similar to *C. dejdaranensis*, but differs in strongly sculptured gaster, petiolar node of major workers trapezoidal in profile, and less convex propodeum.

#### Description.

**Figure 19.**
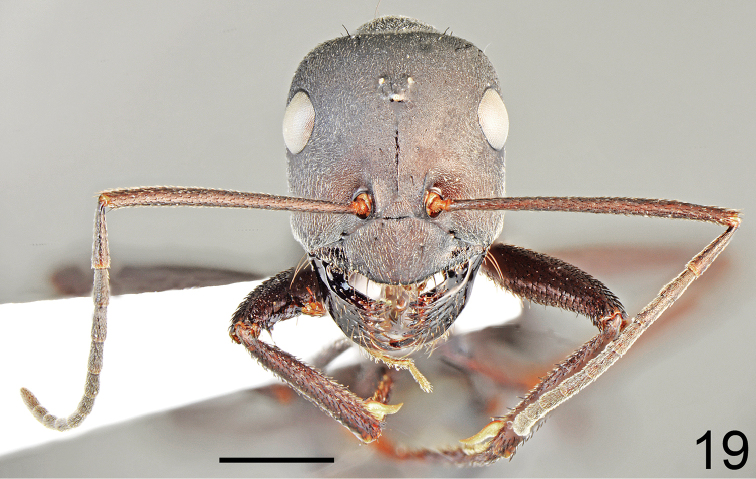
Major worker of *Cataglyphis dejdaranensis* sp. nov., head and antennae. Scale bar 1 mm.

Major worker (n = 6): ***Measurements.***HL: 2.197 (2.07–2.14); HW: 1.970 (1.85–2.12); SL: 2.413 (2.25–2.64); PW: 1.350 (1.25–1.46); PRL: 1.178 (1.08–1.32); PRW: 0.998 (0.93–1.09); PTH: 0.877 (0.78–0.94); PTW: 0.452 (0.42–0.49); WL: 3.263 (3.05–3.48); HFL: 3.402 (3.13–3.69); CI: 1.117 (1.085–1.141); SI: 1.098 (1.064–1.131); PI: 1.945 (1.694–2.163); FI: 1.042 (1.024–1.098). ***Color.*** Head, mesosoma and gaster completely black or head anteriorly, and pronotum and mesosoma on lateral sides with indistinct brownish black spots of diffused borders. Legs completely black or black-brown with tarsi sometimes slightly paler than femora and tibiae. Antennae completely black (Figs 17–19, 23, 24). ***Head.*** Square; approximately 1.12× as long as wide; sides below eyes almost parallel, above eyes gently convex, posterior margin almost straight (Fig. 19). Anterior margin of the clypeus convex; without central impression; with a row of short black setae, and eight longer black setae, the longest as long as 0.6 length of clypeus; clypeal plate with a pair of long and black setae centrally and a pair of similar setae basally. Clypeus opalescent and densely microreticulated; covered with sparse, short and adpressed hairs. Eyes large and oval; approximately 1.2× as long as wide. Frontal carinae short, not extending beyond frontal lobes; interocular area with thin shiny line and two or three long black setae along each of its sides. Antennal fossa shallow, opalescent, densely microreticulated. Head opalescent and densely microreticulated; covered with sparse, short, adpressed hairs (Fig. 19). Ocellar region with group of 2–4 moderately long black setae; posterior angles with two or three long and 1–3 short black setae; rest of frontal and lateral faces of head without erect setae; ventral side of the head with a dozen white to brown setae. Antennal scape long; in frontal view straight; 1.1× as long as width of the head; its base without tooth; from base to apex slightly and gradually widening; funiculus long; pedicel elongated, approximately 0.85× as long as segments II and III combined and 1.7× as long as segment II (Fig. 19). Surface of scape densely microsculptured, opalescent; covered with thick, dense, decumbent setae. Mandibles rounded; basally smooth and shiny; apical half with deep grooves; surface shiny with several long white setae; masticatory margin with four large teeth. ***Mesosoma.*** Long, 2.2× as long as wide; metanotal groove shallow. Pronotum convex on sides. In lateral view promesonotum slightly arched in profile, propodeum positioned lower than promesonotum; distinctly convex in lateral view (Figs 24, 25). Mesosoma opalescent and densely microreticulated; covered with sparse, short and adpressed hairs. Pronotum posteriorly with 2–4 moderately long and black setae; and anteriorly with 2–4 short black setae; sometimes setae broken or missing; mesonotum with up to two moderately long and black setae close to the anterior margin and usually two black setae close to the median groove; propodeum apically with 1–4 short, black setae; sometimes mesonotal and propodeal setae broken. ***Petiole.*** In form of knob; its anterior face distinctly convex; posterior face only slightly convex and dorsum regularly rounded; peduncle short. Surface opalescent and densely microreticulated; covered with dense, short, adpressed hairs; dorsum of knob with 3–5 very short, black, erect setae. ***Gaster.*** Finely microreticulated and moderately shiny; gastral segment I and lateral sides of remaining segments appear distinctly shinier than remaining parts of gaster. Whole surface of gaster with short, sparse, adpressed hairs; tergites I and II without erect setae; tergite III with one or two long black setae centrally and close to anterior margin, and up to one setae at lateral margin; tergite IV in younger specimens with four long black setae on each side; in older specimens setae usually broken (Fig. 24). Each of gastral sternites with three or four long, black, erect setae. ***Legs.*** Dorsal and lateral surfaces of femora and tibiae covered with thick, dense, black decumbent setae; no white adpressed setae on surface between black setae. Ventral surfaces of femora and tibiae with numerous long, black, suberect to erect setae.

Minor worker (n = 4): ***Measurements.***HL: 1.575 (1.39–1.71); HW: 1.373 (1.19–1.53); SL: 1.593 (1.35–1.85); PW: 0.948 (0.83–1.04); PRL: 0.860 (0.76–0.96); PRW: 0.700 (0.62–0.77); PTH: 0.595 (0.54–0.65); PTW: 0.330 (0.28–0.35); WL: 2.303 (2.02–2.48); HFL: 2.185 (1.81–2.53); CI: 1.149 (1.118–1.168); SI: 1.009 (0.971–1.082); PI: 1.808 (1.647–1.929); FI: 0.946 (0.896–1.020).

***Color.*** Slightly paler than major workers; head, mesosoma and gaster mostly brown to dark brown; upper part of head, propodeum and gaster usually blackish brown but never black; transition from brown to blackish brown diffused. Antennae and legs brown to almost black, legs brown to dark brown (Figs 23, 24). ***Body.*** Morphological characters similar to these of major worker except petiole which appears as a very thick scale; approximately 2× as high as broad, with anterior surface only slightly convex and posterior surface flat, and with only rudiment of peduncle (Fig. 21). Dorsal surface of gastral tergites I and II without erect setae; tergite III without or with only a pair of erect setae; tergite IV with two or four setae. Sternites setose as in major worker. Legs generally similarly setose as in major workers except fewer suberect and erect black setae on ventral sides of femora and tibiae. Head slightly longer and scape slightly shorter than in major workers.

**Figures 20–22.**
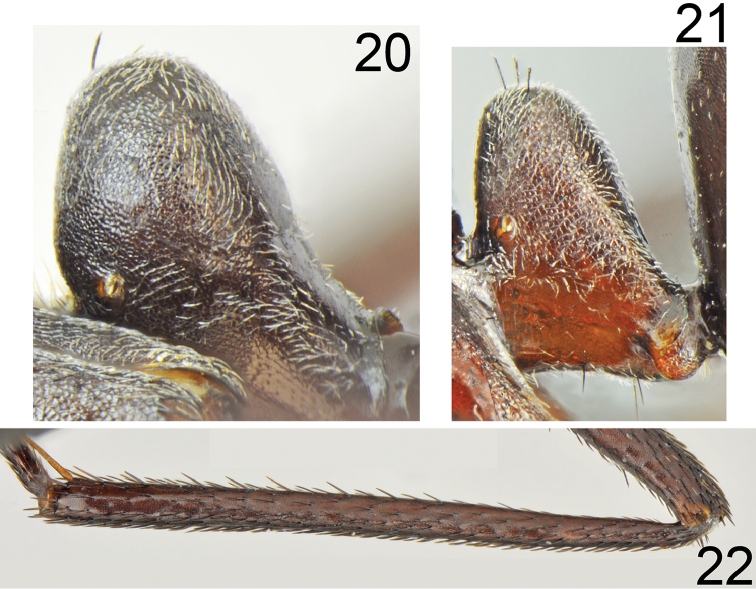
*Cataglyphis dejdaranensis* sp. nov. **20** petiole of major worker **21** petiole of minor worker **22** hind tibia (not in scale).

**Figures 23, 24.**
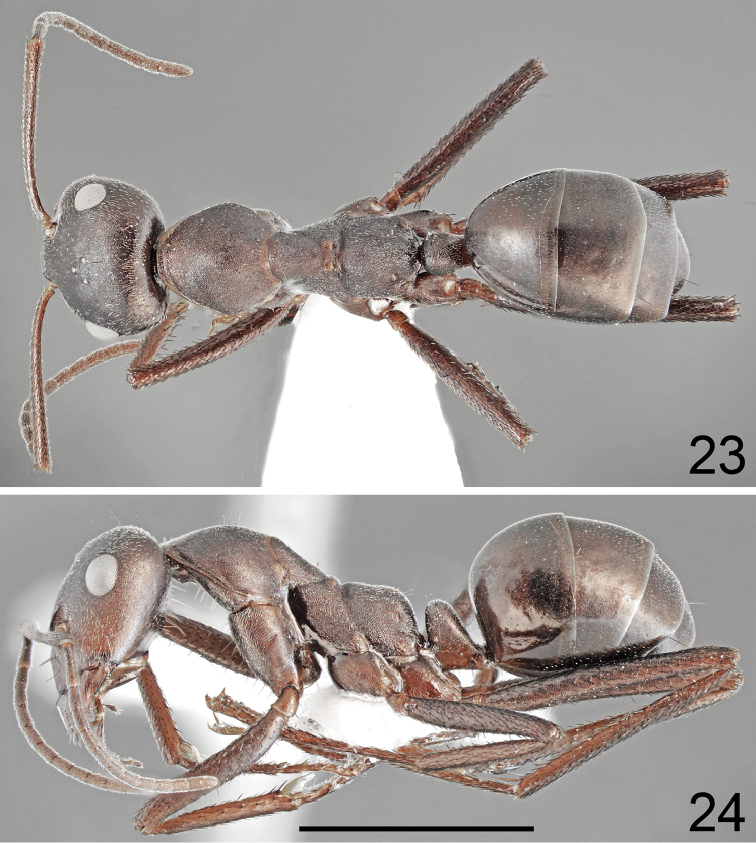
Minor worker of *Cataglyphis dejdaranensis* sp. nov. **23** dorsal **24** lateral. Scale bar 2 mm.

#### Biology.

Little known. On the locality Cheri, specimens were collected in a mountain grazing area overgrown by grass, and on the locality Dejdaran Valley ants were found on mountain pastures with scant vegetation (Fig. 26). Nests were located under large stones. Both collecting sites were placed on high altitude: 2319 m and 2778 m.

#### Etymology.

The species name *dejdaranensis* is a feminine Latin adjective in the nominative case and refers to Dejdaran Valley, where one of the specimens of this species was collected.

**Figures 25, 26.**
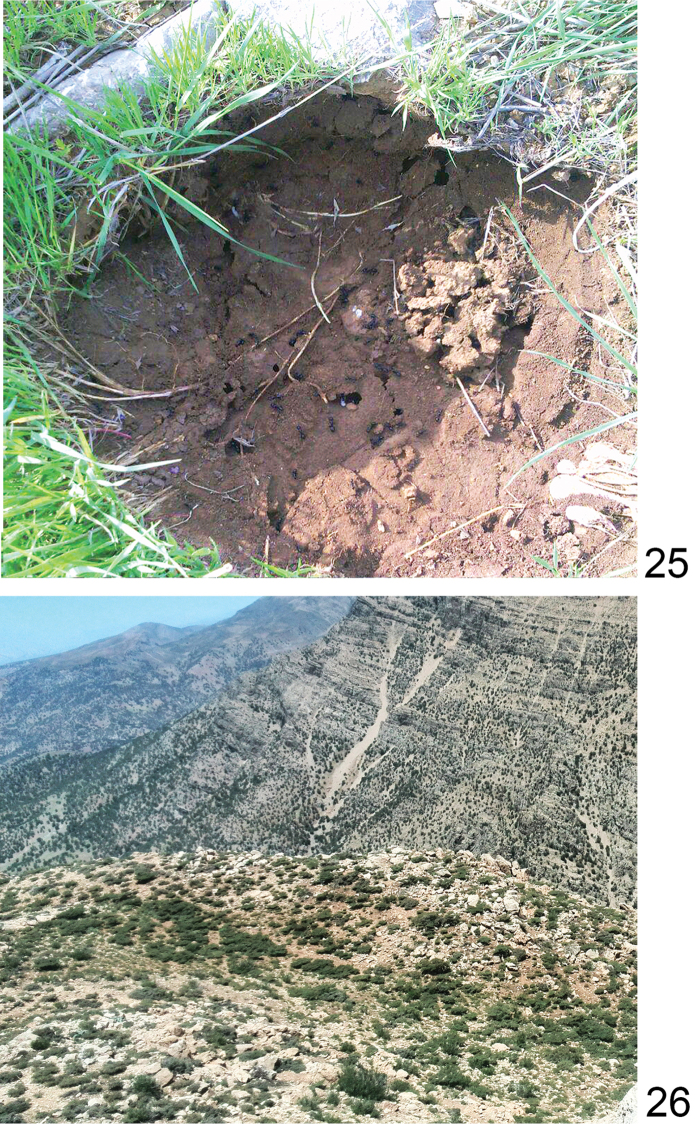
Nest of *Cataglyphis fritillariae* under a large stone at the Dashte laleh site (**25)** Locality Cheri (**26)**.

**Figures 27–34.**
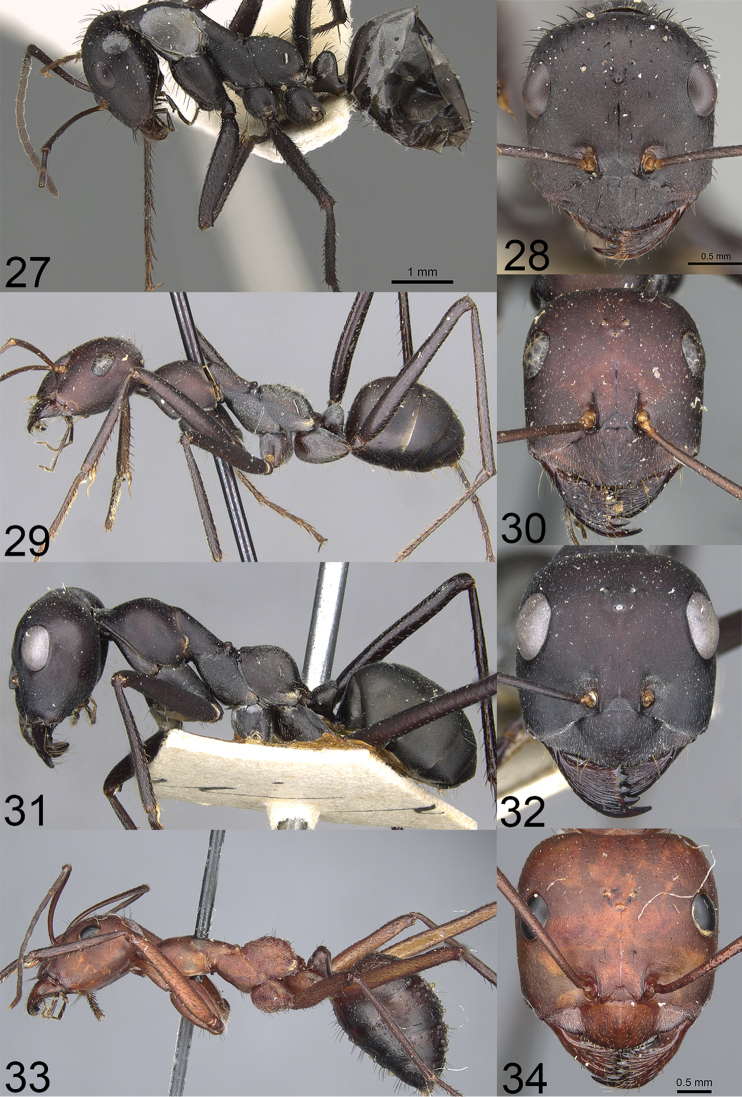
Photographs of members of the *Cataglyphis altisquamis* species group. *C. asiriensis* Collingwood **27** lateral **28** head (Michele Esposito, from www.antweb.org), *C. bucharica* Emery **29** lateral **30** head (Zach Lieberman, from www.antweb.org), *C. foreli* (Ruzsky) **31** lateral **32** head (Zach Lieberman, from www.antweb.org), *C. kurdistanica* Pisarski **33** lateral **34** head (Kate Martynova, from www.antweb.org).

## Comments

All three new Iranian species belong to the group of large, polymorphic *Cataglyphis*. Based on their morphology, they should be assigned to the *Cataglyphis altisquamis* group, sensu Agosti (1990). Herein, we present a modified version of the key to the Asian *Cataglyphis* (Radchenko 1998). The key was modified to accommodate the three new species and *C. asiriensis*, which was not included in the original version. We also decided not to refer to figures available in Radchenko’s paper but provide photographs of type specimens of species which are included in the key (except photographs of *C. oxiana* Arnol’di and *C. piligera* Arnol’di that were not available).

In the key to the Asian *Cataglyphis altisquamis* species group proposed by Radchenko (1998) all four species run to couplet 39 and the key is modified as follow:

**Table:** 

1	[39 in Radchenko 1998]. Petiole wide-cuneiform or conical; if petiole slightly node-shaped, then eyes large, as long as, or 0.8× as long as genae, and body uniformly black or black-brown	**2**
–	Petiole node-shaped, eyes small less than 0.8× as long as genae, and body never uniformly black to black-brown	18 [in Radchenko 1998]
2	Surface of femora and tibiae covered with black, thick, and decumbent setae	**3**
–	Surface of femora and tibiae not covered with a black, thick, and decumbent setae	**7**
3	Soldier caste present, soldiers have saber-shaped mandibles with blunt denticles along their inner margin. Head and mesosoma yellowish red, gaster red-brown to dark brown, posterior margin of head with black, erect setae (Figs 33, 34). Turkey, Iraq	***C. kurdistanica* Pisarski, 1965**
–	Soldier caste absent. Head, mesosoma and gaster brown to black, if red- brown then posterior margin of head without black, erect setae (Figs 1, 2, 9, 10, 15–18, 22, 24)	**4**
4	Mesonotum in major workers distinctly bicolored, head, mesosoma and petiole reddish (Figs 1, 2), petiole in both, major and minor workers conical (Figs 4, 5). Iran	***C. bazoftensis* sp. nov.**
–	Mesonotum in both major and minor workers uniformly brown to black (Figs 9, 10, 15–18, 22, 24), petiole in major workers trapezoidal or knob-shaped, in minor workers in form of a thick squama (Figs 12, 13, 20, 21)	**5**
5	Both dorsal and ventral surfaces of femora and tibiae with a row of long, black, and spiniform setae, mesosoma with thick and black setae (Figs 27, 28) Saudi Arabia	***C. asiriensis* Collingwood, 1985**
–	Only ventral surfaces of femora and tibiae with row of long, black, and spiniform setae, mesosoma with fewer and thinner setae (Figs 9, 15, 17, 24)	**6**
6	Gaster dull (Figs 9, 15). Petiole in major worker trapezoidal in profile (Fig. 12). Iran	***C. fritillariae* sp. nov.**
–	At least sides of gastral tergite I with relatively shiny area (Figs 17, 24). Petiole in major worker knob-shaped in profile (Fig. 20). Iran	***C. dejdaranensis* sp. nov.**
7	Mesosoma and posterior margin of head with numerous erect setae (Figs 29, 30)	**8**
–	Mesosoma and posterior margin of head without or with sparse erect hairs (Figs 31, 32). Body black or black-brown	**9**
8	Propodeal dorsum distinctly longer than its declivity in profile. Petiole high, narrow-cuneiform, with weakly convex anterior surface, about as high as propodeum. Head and mesosoma red to red-brown (posterior half of thorax sometimes dark brown); gaster dark brown to black. Mountains of Uzbekistan and Tajikistan; Afghanistan and N Iran	***C. bucharica* Emery**
–	Propodeal dorsum as long as its declivity in profile. Petiole cuneiform, with strongly convex anterior surface, clearly lower than propodeum. Body uniformly dark brown to black. Turkmenistan and Uzbekistan	***C. piligera* Arnol’di, 1964**
9	Eyes small, 0.6–0.5× length of genae. Middle East	***C. altisquamis* (André, 1881)**
–	Eyes large, 0.8–1× as long as genae	**10**
10	Petiole low, nearly node-shaped, posterior margin of head strongly convex, rounded. Turkmenistan and Uzbekistan	***C. oxiana* Arnol’di, 1964**
–	Petiole comparatively higher, broad- cuneiform. In large workers, posterior margin of head straight or slightly concave. Turkmenistan, Iran	***C. foreli* (Ruzsky, 1903)**

## Supplementary Material

XML Treatment for Cataglyphis bazoftensis

XML Treatment for Cataglyphis fritillariae

XML Treatment for Cataglyphis dejdaranensis

## References

[B1] Agosti D (1990) Review and reclassification of *Cataglyphis* (Hymenoptera, Formicidae). Journal of Natural History 24: 1457–1506. 10.1080/00222939000770851

[B2] Amor F Ortega P (2014) *Cataglyphis tartessica* sp. n., a new ant species (Hymenoptera: Formicidae) in south-western Spain. Myrmecological News 19: 125–132. 10.5281/zenodo.16346

[B3] Arakelian GR (1994) Fauna of the Republic of Armenia. Hymenopterous insects. Ants (Formicidae). Gitutium, Erevan, 153 pp. [In Russian]

[B4] Arnol’di KV Dlussky GM (1978) Superfam. Formicoidea. 1. Fam. Formicidae – ants. In: Medvedev GS (Ed.) Keys to the insects of the European part of the USSR. Vol. 3. Hymenoptera. Part 1.Opredeliteli Faune SSSR 119: 519–556. [In Russian]

[B5] Atanassov N Dlussky GM (1992) Fauna of Bulgaria. Hymenoptera, Formicidae. Fauna of Bulgaria 22: 1–310.

[B6] Bolton B (2020) An online catalog of the ants of the world. https://antcat.org [accessed 06 September 2020]

[B7] Boulay R Carro F Soriguer RC Cerdá X (2007) Synchrony between fruit maturation and effective dispersers’ foraging activity increases seed protection against seed predators. Proceedings of the Royal Society of London B 274: 2515–2522. 10.1098/rspb.2007.0594 PMC2275878 17698486

[B8] Brown Jr WL (2000) Diversity of ants. In: Agosti D, Majer J, Alonso E, Schultz TR (Eds) Ants: standard methods for measuring and monitoring biodiversity. Biological diversity hand book series. Smithsonian Institution Press, Washington, DC, 280 pp.

[B9] Cagniant H (2009) Le genre *Cataglyphis* Foerster, 1850 au Maroc (Hyménoptères Formicidae). Orsis 24: 41–71. https://www.antwiki.org/wiki/images/c/ca/Cagniant_Cataglyphis_2009.pdf

[B10] Chang YD He DH (2002) Three new species of the genus *Cataglyphis* Foerster from Northwest China (Hymenoptera: Formicidae: Formicinae). Zoological Research 23: 61–64. [In Chinese]

[B11] Collingwood CA (1978) A provisional list of Iberian Formicidae with a key to the worker caste (Hym. Aculeata). EOS. Revista Española de Entomología 52: 65–95. 10.5281/zenodo.26690

[B12] Collingwood CA Agosti D (1996) Formicidae (Insecta: Hymenoptera) of Saudi Arabia (Part 2). Fauna of Saudi Arabia 15: 300–385. https://antcat.org/references/130931

[B13] Collingwood C Prince A (1998) A guide to ants of continental Portugal (Hymenoptera: Formicidae). Boletim da Sociedade Portuguesa de Entomologia. Suplemento 5: 1–49. 10.5281/zenodo.1244612

[B14] Collingwood C Agosti D Sharaf MR van Harten A (2011) Order Hymenoptera, family Formicidae. Arthropod fauna of the UAE 4: 405–474. 10.5281/zenodo.1168586

[B15] Crawley WC (1920) Ants from Mesopotamia and north-west Persia (concluded). The Entomologist’s Record and Journal of Variation 32: 177–179. 10.5281/zenodo.15001

[B16] Dlussky GM Soyunov OS Zabelin SI (1990) Ants of Turkmenistan. Ylym Press, Ashkabad, 273 pp. [In Russian]

[B17] Emery C (1906) Rassegna critica delle specie paleartiche del genere Myrmecocystus. Memorie della Reale Accademia delle Scienze dell’Istituto di Bologna (6)3: 47–61. 10.5281/zenodo.25506

[B18] Evenhuis N (2020) The insect and spider collections of the world website. http://hbs.bishopmuseum.org/codens/ [accessed 10 Augst 2020]

[B19] Eyer PA Seltzer R Reiner-Brodetzki Hefetz A (2016) An integrative approach to untangling species delimitation in the *Cataglyphis bicolor* desert ant complex in Israel. Molecular Phylogenetics and Evolution 115: 128–139. 10.1016/j.ympev.2017.07.024 28774791

[B20] Forel A (1904) Note sur les fourmis du Musée Zoologique de l’Académie Impériale des Sciences à St. Pétersbourg. Ezhegodnik Zoologicheskago Muzeya Imperatorskoi Akadernii Nauk 8: 368–388. 10.5281/zenodo.25586

[B21] Ghahari H Collingwood CA (2011) A study on the ants (Hymenoptera: Formicidae) of southern Iran. Calodema 176: 1–5.

[B22] Ghahari H Collingwood CA Havaskary M Ostovan H Samin N (2011) A contribution to the knowledge of ants (Hymenoptera: Formicidae) from the Arasbaran biosphere reserve and vicinity, Northwestern Iran. Jordan Journal of Agricultural Sciences 7(3): 558–563. https://www.antwiki.org/wiki/images/1/11/Ghahari_et_al_2011.pdf

[B23] Ghahari H Collingwood CA Tabari M Ostovan H (2009) Faunistic notes on Formicidae (Insecta: Hymenoptera) of rice fields and surrounding grasslands in northern Iran. Munis Entomology & Zoology Journal 4(1): 184–189. https://www.munisentzool.org/yayin/vol4/issue1/184–189.pdf

[B24] Herrera CM Herrera J Espadaler X (1984) Nectar thievery by ants from southern Spanish insect-pollinated flowers. Insectes Sociaux 31: 142–154. 10.1007/BF02232711

[B25] Hulme PE (1997) Post-dispersal seed predation and the establishment of vertebrate dispersed plants in Mediterranean scrublands. Oecologia 111: 91–98. 10.1007/s004420050212 28307510

[B26] Ionescu A Eyer P-A (2015) Notes on *Cataglyphis* Foerster, 1850 of the bicolor species group in Israel, with description of a new species (Hymenoptera: Formicidae). Israel Journal of Entomology 46: 109–131. 10.5281/zenodo.221456

[B27] Janicki J Narula N Ziegler M Guénard B Economo EP (2016) Visualizing and interacting with large-volume biodiversity data using client-server web-mapping applications: The design and implementation of antmaps.org. Ecological Informatics 32: 185–193. 10.1016/j.ecoinf.2016.02.006

[B28] Karavaiev V (1924) Zur Systematik der paläarktischen Myrmecocystus (Formicidae), nebst einigen biologischen Notizen. Konowia 3: 301–308. 10.5281/zenodo.25958

[B29] Lenoir A Aron S Cerdá X Hefetz A (2010) *Cataglyphis* desert ants: a good model for evolutionary biology in Darwin’s anniversary year—a review. Israel Journal of Entomology 39: 1–32. http://digital.csic.es/bitstream/10261/65135/1/israel.pdf

[B30] Moradloo S Fard RN Rad SP Taylor B (2015) Records of ants (Hymenoptera: Formicidae) from Northern Iran. Zoology in the Middle East 2015: 1–6. 10.1080/09397140.2015.1020611

[B31] Paknia O Radchenko A Alipanah H Pfeiffer M (2008) A preliminary check list of the ants (Hymenoptera: Formicidae) of Iran. Myrmecological News 151–159. http://antbase.org/ants/publications/21820/21820.pdf

[B32] Paknia O Radchenko A Pfeiffer M (2009) New records of ants (Hymenoptera, Formicidae) from Iran. Asian Myrmecology 3: 29–38.

[B33] Pisarski B (1965) Les fourmis du genre *Cataglyphis* Foerst. en Irak (Hymenoptera, Formicidae). Bulletin de l’Académie Polonaise des Sciences. Série des Sciences Biologiques 13: 417–422. 10.5281/zenodo.25692

[B34] Rad SP Taylor B Torabi R Aram E Abolfathi Afshari Borjali F Ghatei M Hediary F Jazini F Kiah VH Mahmoudi Z Sararyan F Seiri M (2018) Further records of ants (Hymenoptera: Formicidae) from Iran. Zoology in the Middle East 2015: 1–15.

[B35] Radchenko AG (1997) Review of ants of the genus *Cataglyphis* Foerster (Hymenoptera, Formicidae) of Asia. Entomologicheskoe Obozrenie 76: 424–442. [In Russian]

[B36] Radchenko AG (1998) A key to the ants of the genus *Cataglyphis* Foerster (Hymenoptera, Formicidae) of Asia. Entomologicheskoe Obozrenie 77: 502–508. [In Russian]

[B37] Radchenko AG Paknia O (2010) Two new species of the genus *Cataglyphis* Foerster, 1850 (Hymenoptera: Formicidae) from Iran. Annales Zoologici 60: 69–76. 10.3161/000345410X499533

[B38] Salata S Borowiec L (2018) Taxonomic and faunistic notes on Greek ants (Hymenoptera: Formicidae). Annals of the Upper Silesian Museum in Bytom Entomology 27(online 008): 1–51. 10.5281/zenodo.2199191

[B39] Santschi F (1929) Étude sur les *Cataglyphis*. Revue Suisse de Zoologie 36: 25–70. 10.5962/bhl.part.117626

[B40] Seifert B (2018) The ants of Central and North Europe. lutra Verlags- und Vertriebsgesellschaft, Tauer, Germany, 407 pp.

[B41] Sharaf MR Collingwood CA Aldawood AS (2015) Notes on the ant genus *Cataglyphis* Foerster, 1850 (Hymenoptera, Formicidae) in the Arabian Peninsula with description of a new species and a key to species of the *C. pallida*-group. ZooKeys 545: 101–117. 10.3897/zookeys.545.6308 PMC4714370 26798297

[B42] Wehner R (2020) Desert navigator. The journey of an ant. The Belknap press of Harvard University Press, Cambridge, Massachusetts, 392 pp 10.4159/9780674247918

[B43] Wilson EO (1955) A monographic revision of the ant genus *Lasius*. Bulletin of the Museum of Comparative Zoology 113: 1–201. 10.5281/zenodo.25290

